# Advancements in Pathogen Detection: Argonaute-Based Nucleic Acid Detection Technology

**DOI:** 10.3390/pathogens14060554

**Published:** 2025-06-02

**Authors:** Meng Hong, Guodi Wu, Yanli Ren, Shanshan Wu, Haihong Zhu, Zhi Chen

**Affiliations:** State Key Laboratory for Diagnosis and Treatment of Infectious Diseases, National Clinical Research Center for Infectious Diseases, National Medical Center for Infectious Diseases, Collaborative Innovation Center for Diagnosis and Treatment of Infectious Diseases, The First Affiliated Hospital, Zhejiang University School of Medicine, Hangzhou 310000, China; hongmeng@zju.edu.cn (M.H.); wuguodi911@163.com (G.W.); renren2004a@163.com (Y.R.); wushanshan08@zju.edu.cn (S.W.)

**Keywords:** Argonaute, pathogen detection, nucleic acid testing

## Abstract

In recent years, global public health security has encountered significant challenges, with infectious diseases accounting for approximately 25% of global mortality annually. The worldwide pandemic instigated by the novel coronavirus, alongside the persistent threats posed by Ebola, influenza, and multidrug-resistant bacteria, has severely compromised human health, economic development, and social stability. Within this context, the development of rapid and precise pathogen detection technologies has emerged as a critical frontline defense for epidemic prevention and control, serving as a pivotal component in the implementation of the “early detection, early isolation, and early treatment” strategy. The Argonaute (Ago) protein, recognized as a programmable and target-specific activated nuclease, has demonstrated substantial potential in the realm of nucleic acid detection due to its distinctive biological properties, garnering considerable attention. In this study, we delineate the structural characteristics of Ago proteins and elucidate the mechanism underlying their nuclease activity. Furthermore, we review the principles of nucleic acid detection based on Argonaute and provide a comprehensive analysis of recent advancements in related detection systems. Additionally, we compare the advantages of detection based on Argonaute with other detection methodologies. Through a comprehensive analysis, we aim to provide a robust theoretical foundation and an advanced technical reference for the development of new-generation nucleic acid detection platforms with high sensitivity and high specificity.

## 1. Introduction

In recent years, the threat of infectious diseases such as the novel coronavirus [1], Zika virus [2], Ebola virus [3], dengue fever, influenza virus, and multidrug-resistant bacteria has continued to increase, posing a severe test to the global health system, economic development, and social stability. According to the latest statistics, there are 60 million deaths worldwide every year, of which at least 25% are caused by infectious diseases [4]. Within this context, the development of rapid and precise pathogen detection technologies has emerged as a critical frontline defense for epidemic prevention and control, serving as a pivotal component in the implementation of the “early detection, early isolation, and early treatment” strategy.

In comparison to conventional pathogen diagnostic methods, such as microbial culture, nucleic acid detection technologies demonstrate enhanced sensitivity and specificity, coupled with reduced detection times, typically around two hours. Presently, quantitative polymerase chain reaction (qPCR) and gene sequencing technologies [5] are esteemed as the “gold standards” in nucleic acid detection. Nonetheless, these methodologies necessitate costly diagnostic equipment, highly trained personnel, and well-equipped clinical gene amplification laboratories [6]. Furthermore, qPCR exhibits limited efficacy in identifying single-nucleotide polymorphisms. To overcome these constraints, nucleic acid diagnostic technologies leveraging gene-editing proteins, including CRISPR/Cas and Argonaute (Ago), have been developed in recent years. The CRISPR-Cas detection system facilitates the precise targeting and cleavage of specific gene sequences by directing crRNA/gRNA to protospacer adjacent motif (PAM) sites. Additionally, this system induces potent nonspecific “collateral cleavage” activity, which indiscriminately cleaves single-stranded DNA/RNA sequences in proximity to the target gene. Researchers can attain more highly specific and sensitive detection of target nucleic acids by utilizing the signal amplification capabilities of the CRISPR/Cas system’s collateral cleavage activity, selecting suitable target genes, and using effective signal reporters. Nonetheless, this approach faces challenges in achieving the multiplex detection of multiple target genes within a single reaction system [7], thereby limiting its applicability across diverse scenarios. Ago proteins, due to their distinctive nucleic acid binding and cleavage capabilities, facilitate the specific recognition of single-nucleotide mutations and enable the multiplex detection of pathogens. This offers a novel technological pathway for pathogen diagnostics in the era of precision medicine, providing essential technical resources for addressing potential future public health crises. This article offers a comprehensive review of the structural features of Ago proteins and their mechanisms for nucleic acid recognition, presents a review of the latest advancements and applications of related technologies in pathogen detection, and explores the challenges encountered in practical applications. Additionally, it offers a forward-looking perspective on future developmental directions, aiming to provide insights into the field.

## 2. Mechanism of Nucleic Acid Recognition Mediated by Argonaute Proteins

### 2.1. Argonaute

In 1998, the Ago protein was first discovered in a study of *Arabidopsis thaliana* mutants [8]. This protein family is widely present in various organisms in nature [9]. Serving as a key element in the RNA interference (RNAi) system, Ago proteins bind to small interfering RNA (siRNA), PIWI-interacting RNA (piRNA), and microRNA (miRNA) [10] to form the RNA-induced silencing complex, which subsequently degrades target RNA to maintain gene silencing [11,12]. This process plays a critical role in cellular defense against viral infections [13]. Ago proteins can be classified into two groups: prokaryotic Argonaute proteins (pAgos) and eukaryotic Argonaute proteins (eAgos). Among them, eAgos exhibit conserved structural features [9], including an N-terminal domain, PAZ (PIWI–Argonaute–Zwille), MID (middle domain), and PIWI domain. Among the four structural domains, the N-terminal domain exhibits the lowest degree of conservation and may play a facilitative role in double-stranded RNA (dsRNA) unwinding and strand separation [14]. The PAZ and MID domains are key regions for binding the guide strand, responsible for the specific recognition of the 3′ and 5′ ends of the guide strand, respectively [15]. The PIWI domain encompasses a conserved catalytic tetrad, Asp-Glu-Asp-X, in which the X position may be occupied by an aspartate, histidine, or lysine residue. This configuration facilitates the coordination of divalent cations, thereby initiating nucleolytic cleavage [16]. In contrast, pAgos display greater structural diversity and can be classified into three categories based on domain characteristics: short pAgos, long pAgos, and PIWI-domain-containing proteins with conserved R and E residues (PIWI-RE) [17]. Currently, well-studied pAgos include CbAgo from *Clostridium butyricum*, TtAgo from *Thermus thermophilus,* PfAgo from *Pyrococcus furiosus*, and RsAgo from *Rhodobacter sphaeroides*. These pAgos primarily use DNA as the guide strand to target DNA or RNA strands (some specifically target DNA strands only) [18]. Notably, although some pAgos exhibit single-stranded RNA cleavage activity similar to eAgos, their unique DNA-specific cleavage capabilities and DNA interference properties [19] have opened new avenues for their application in molecular detection. The nucleic acid cleavage process mediated by pAgos does not require the participation of PAM sequences, providing a unique advantage for the application of Ago proteins in in vitro nucleic acid detection. By exploiting this characteristic, researchers have crafted a variety of novel Ago-mediated nucleic acid detection technologies, bringing new opportunities for advancement in the field of molecular diagnostics.

### 2.2. Fundamental Principles of Nucleic Acid Cleavage by Argonaute Proteins

**Figure 1:**
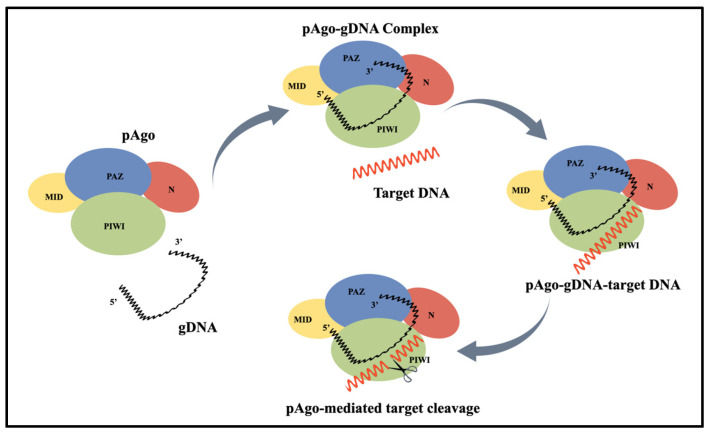
Schematic of pAgo-mediated single-stranded target DNA cleavage. PIWI, MID, PAZ, and N are the domains of pfAgo.

First, the PAZ and MID domains of the pAgo protein bind to the 3′ and 5′ ends of the single-stranded guide DNA (gDNA), respectively, forming a pAgo-guide binary complex. The gDNA within the complex specifically recognizes the target DNA (tDNA), and upon binding, the endonuclease activity of the pAgo protein is activated, enabling targeted cleavage of the tDNA (Figure 1). The phosphodiester bond of the tDNA is broken at the typical site between the 10th and 11th nucleotides from the 5′ end of the gDNA if the guide strand is a 5′-P strand [20,21]. If the guide strand is a 5′-OH strand, a shift of 1–2 nucleotides may occur at the cleavage site [22]. With only one active site, the Ago protein is limited to cleaving one DNA strand at a time. To cleave double-stranded DNA, two guide strands targeting complementary regions of the DNA must be added to the system.

### 2.3. Nucleic Acid Detection Technology Based on Ago Proteins

Argonaute proteins specifically recognize and bind to target DNA or RNA sequences, mediating precise cleavage through their endonuclease activity. This unique property confers significant potential advantages for in vitro diagnostic applications. Below, we summarize several representative Ago-protein-based nucleic acid detection technologies and provide a comprehensive evaluation of these approaches (Table 1).

#### 2.3.1. PfAgo-Mediated Nucleic Acid Detection (PAND)

**Figure 2:**
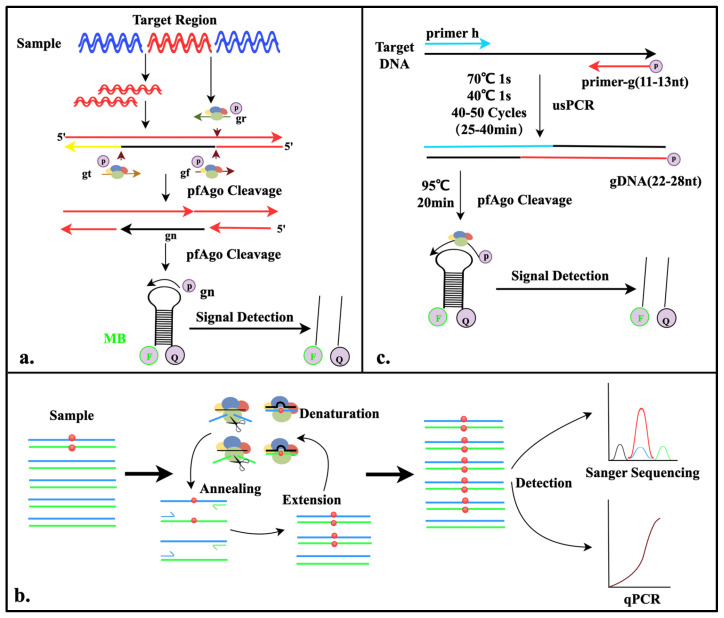
Diagram of the mechanisms involved in the study (I). (**a**). Schematic illustration of PAND detection system. Sequences of three 5′-phosphorylated single-stranded DNA guides (gr, gt, and gf) and newly generated single-stranded DNA (gn). (**b**). Schematic illustration of A-Star detection system. Black lines represent gDNA and blue and green lines represent forward and reverse strands of target dsDNA, respectively. Red dots indicate the mutated nucleotides of SNV targets. (**c**). Schematic illustration of USPCRP detection system. The 5′-phosphorylated primer (primer-g), another primer (primer-h), and molecular beacons (MBs) are highlighted in different colors.

In 2019, He et al. developed the first nucleic acid detection platform—PAND—based on a thermophilic Argonaute from *Pyrococcus furiosus* (PfAgo) [23]. This method initially involves the amplification of target fragments through PCR. The PfAgo-gDNA complex then specifically cleaves the amplified double-stranded DNA (dsDNA) to generate short single-stranded DNA (ssDNA). These newly generated ssDNA molecules can serve as new gDNA to bind with PfAgo again, activating a follow-up round of cleavage for downstream fluorescent probes. This process ultimately releases fluorescent signals, which are used as specific markers to detect the presence of target nucleic acid. By integrating PCR technology, this detection platform achieved attomolar-level sensitivity (10 aM) for DNA detection and enabled the simultaneous multiplex detection of DNA across five channels using only a single Argonaute protein (Figure 2a).

#### 2.3.2. Ago-Directed Specific Target Enrichment and Detection (A-Star)

Liu et al. developed the A-Star detection system, which enables the precise identification of single-nucleotide variation (SNV) target sequences through the action of Argonaute nucleases, thereby achieving “one-tube” PCR-based multiplex nucleic acid enrichment and detection. Guided by gDNA, PfAgo performs precise cleavage, and through a cleverly designed guide strand mismatch strategy, it achieves the single-base discrimination of target nucleic acids. This system establishes a PfAgo-PCR coupling mechanism, where PfAgo selectively cleaves wild-type genes during the PCR denaturation step (94 °C), while the retained mutant genes undergo cyclic amplification during the PCR annealing (~58 °C) and extension (~72 °C) steps. This process enables the ultra-efficient enrichment and accurate detection of low-abundance mutant genes. The enriched samples are compatible with multiple downstream detection techniques, such as Sanger sequencing and qPCR [24] (Figure 2b).

#### 2.3.3. Nucleic Acid Detection via Combination of Ultrashort PCR and *Pyrococcus furiosus* Argonaute (USPCRP)

This detection method employs two primers, each less than 14 nucleotides long, to amplify target DNA through PCR, producing very short DNA fragments of 22–28 base pairs with 5′ phosphate modifications. These products can directly serve as gDNA to guide PfAgo in the specific cleavage of molecular beacons. Due to PfAgo’s lack of sensitivity to gDNA under 14 nucleotides, the amplified primers with 5′ phosphate modifications avoid causing nonspecific cleavage of the fluorescent probes. The detection system established by He et al. simplifies the operation and eliminates the need for additional gDNA. Utilizing just two enzymes, Taq DNA polymerase and PfAgo, it reaches a sensitivity of 10 aM and offers single-base resolution with excellent specificity. This system has been successfully applied to detect viruses in clinical samples [25] (Figure 2c).

#### 2.3.4. TtAgo-Assisted Exponential Isothermal Amplification for Multiplex Detection (TEAM)

**Figure 3:**
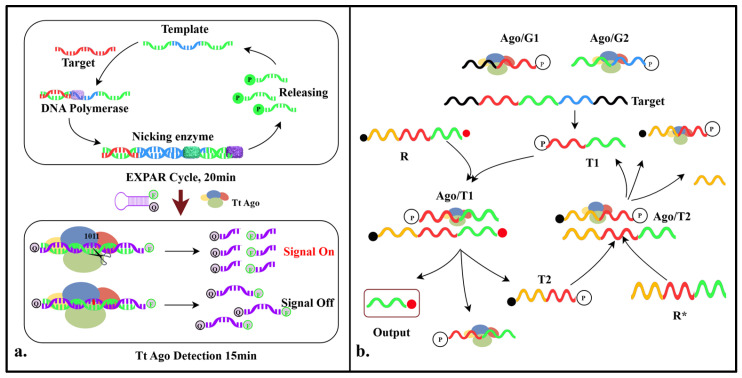
Diagram of the mechanisms involved in the study (II). (**a**). Schematic illustration of TEAM detection system. (**b**). Schematic illustration of ANCA detection system. First, Ago/G1 and Ago/G2 complexes hybridize and cleave target DNA, generating T1. Second, the Ago/T1 complex forms, and it hybridizes and cleaves R, releasing output and T2. Third, the Ago/T2 complex forms, and it hybridizes and cleaves R*, generating T1 and thus completing the ANCA.

Lin et al. developed the TEAM detection system, which integrates the programmability, high specificity, and multi-turnover cleavage mechanism of the TtAgo protein with the Exponential Amplification Reaction (EXPAR) to achieve the efficient and programmable multiplex detection of miRNA. In this system, the process begins with the exponential amplification of the target miRNA by Vent (exo-) DNA polymerase, and then it is specifically cleaved by the Nt.BstNBI when an ssDNA template is available. This process generates a large number of amplicons with 5′-phosphate groups, which can activate the gDNA-guided TtAgo protein to cleave hairpin reporter probes. The cleavage of the reporter probes results in the separation of the fluorophore from the quencher, leading to a significant increase in fluorescence signal. This system not only retains the high amplification efficiency of traditional EXPAR technology but also significantly enhances its specificity and sensitivity, enabling precise discrimination at the single-nucleotide level. The detection sensitivity reaches single-molecule concentrations (attomolar level), and the entire process can be completed in just 30–35 min [26] (Figure 3a).

#### 2.3.5. Artificial Nucleic Acid Circuit with Argonaute Protein (ANCA)

Developed by Jang et al., the ANCA is a DNA detection technology that merges artificial nucleic acid circuits with the cleavage function of Ago proteins to create a highly specific and exponentially signal-amplified positive feedback loop. This reaction is a one-step process and can easily be adapted to detect different targets by altering the nucleic acid recognition sites. The system consists of guide DNA 1 (G1), guide DNA 2 (G2), a reporter gene (R), and a complementary reporter gene sequence (R *). The Ago/G1 and Ago/G2 complexes hybridize with DNA sequences that match the target complementarily, cleaving the corresponding targets to produce short DNA fragments (T1). The Ago/T1 complex, formed with T1 as a guide DNA, can recognize and cleave R, resulting in the release of output and T2 (two short DNA strands). A fluorescent signal is produced by the output, while T2 and the Ago protein work together to recognize and cleave R*, resulting in T1 and completing the DNA cycle. This positive feedback loop allows the cleavage reaction to be repeated when the target DNA is present, and the fluorescence signal is monitored to determine the presence of the target DNA in the sample [27] (Figure 3b).

#### 2.3.6. Multiplex Ago-Based Nucleic Acid Detection System (MULAN)

**Figure 4:**
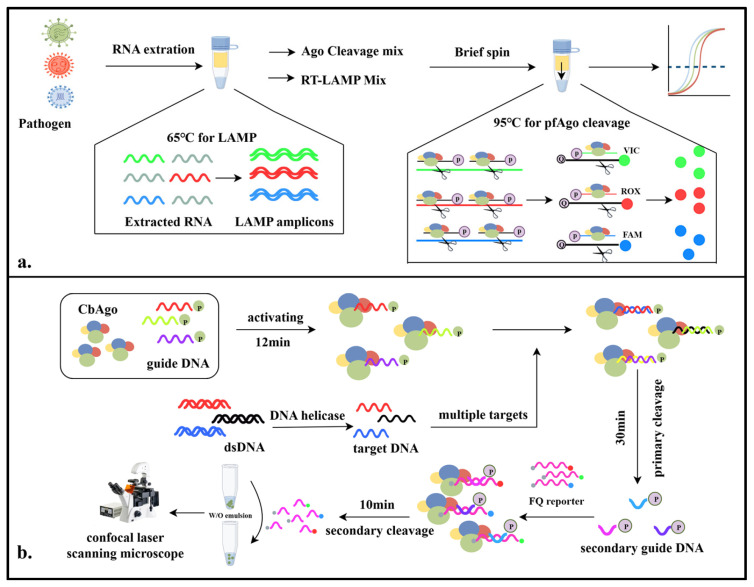
Diagram of the mechanisms involved in the study (III). (**a**). Schematic illustration of MULAN detection system. (**b**). Schematic illustration of PASS detection system.

The MULAN detection system was developed by Ye et al. and leveraged the cascade cleavage mechanism of thermophilic Argonaute enzymes combined with rapid isothermal amplification technology to achieve the highly sensitive, specific, and rapid portable detection of viral samples. Initially, this detection technique utilizes loop-mediated isothermal amplification (LAMP) to rapidly increase the amount of viral nucleic acid samples at 65 °C. The amplified products are then mixed with a thermophilic Argonaute cleavage system. Guided by a pair of gDNAs, the system ingeniously utilizes the cleavage product—sDNA—to mediate the secondary cleavage of fluorescently labeled DNA molecules, generating a specific fluorescent reporter signal for the viral target gene. Studies have shown that the Ago-assisted LAMP isothermal amplification diagnostic system effectively addresses the issue of nonspecific amplification associated with standalone LAMP diagnostics [28] (Figure 4a).

#### 2.3.7. Programmable, Amplification-Free System (PASS)

Wang et al. developed the PASS for detection, which creatively uses CbAgo to develop a programmable detection method for quick nucleic acid quantification without amplification at room temperature. The system uses CbAgo to bind to gDNA and then cleaves the wild-type target DNA that complements the gDNA, transferring fluorescent signals into polydisperse emulsions. The emulsions are then imaged using confocal laser scanning microscopy (CLSM), and the images are processed through deep learning to measure the fluorescence intensity. This detection system enables the application of biosensors at room temperature and makes it feasible to monitor multiplex targets [29]. (Figure 4b).

### 2.4. Strengths and Weaknesses of Ago-Based Detection Technologies

Ago proteins have the capability to recognize target genes independently of the presence of PAM sequences in nucleic acids, thereby offering enhanced flexibility in the selection of target genes for Ago-based nucleic acid detection technologies. Guided by gDNA, Ago proteins demonstrate highly specific sequence recognition abilities, which permits the incorporation of gDNAs targeting various genes within a single reaction system. By utilizing multiple reporter molecules labeled with distinct fluorescent groups, multiplex target detection can be accomplished. Ago proteins are able to target and cleave specific genes only upon binding to gDNA [30], and the high specificity of the interaction between gDNA and target nucleic acids [24] enables the system to detect single-base mutations in viruses. The gDNA recognized by Ago proteins is a short-chain DNA molecule, noted for its high stability, simplicity, and low production costs, which facilitates the production of cost-effective and easily storable reagents, thereby enhancing the accessibility of laboratory operations and field testing. In comparison to Cas nucleases, which necessitate binding to longer RNA molecules, gDNA is shorter and more straightforward to design.

Isothermal amplification technologies, including LAMP, recombinase polymerase amplification (RPA), and rolling circle amplification (RCA), facilitate rapid nucleic acid detection. Nonetheless, these methods often necessitate intricate primer design and the use of numerous primers, which can result in nonspecific amplification, contamination, and reaction systems that are unstable and vulnerable to inhibitors and impurities. By integrating the precise recognition capabilities of Ago proteins with isothermal amplification techniques, the challenge of low specificity inherent in isothermal amplification can be mitigated, thereby substantially enhancing detection efficiency. The combination of the precise recognition attributes of Ago proteins with the signal amplification potential of other technologies allows for the development of highly efficient, cost-effective, and versatile detection systems. For instance, Ago proteins can be utilized in conjunction with lateral flow strips (LFSs) to develop an innovative on-site detection model [31]. Xun et al. created a rapid, scalable, and portable detection system that integrates reverse transcription loop-mediated isothermal amplification with PfAgo and a portable device for the on-site detection of various SARS-CoV-2 targets [32]. Compared to CRISPR-based technologies, PfAgo greatly reduces the detection time, as it cleaves amplification products in just 3–5 min, resulting in the total detection process lasting under 30 min. The MULAN detection system, paired with portable nucleic acid testing devices and consumables, achieves a “one-tube” reaction for amplification and cleavage, effectively minimizing environmental contamination. By integrating colloidal gold immunochromatography and blue-light-excited fluorescence, the results can be visually interpreted, offering a detection sensitivity of 15 copies/reaction. This system demonstrates immense potential for at-home nucleic acid self-testing [28]. The rapid response characteristics of Argonaute-based nucleic acid detection methods, such as the ability to complete detection within 30 min, coupled with their potential for device miniaturization render them highly advantageous for point-of-care testing (POCT) and use in resource-limited settings, such as primary healthcare facilities and field testing stations. These attributes position them as a groundbreaking tool for immediate diagnosis and infectious disease control.

However, Argonaute-based nucleic acid detection technologies also present certain limitations. These systems often necessitate the use of multiple guide DNAs (gDNAs) and involve several procedural steps, which increases both the complexity and cost of experiments. For example, some detection systems require a two-step cleavage process, with each step involving specific gDNA sequences and protein participation, thereby adding to the complexity of the reaction system. Currently, nucleic acid detection technology based on Ago mainly relies on thermophilic Argonaute (PfAgo) and mesophilic Argonaute (TtAgo). To improve the capacity of Ago proteins to detect nucleic acids across a broader temperature spectrum, future research should focus on Ago proteins that function effectively at ambient temperatures, such as those utilizing *Clostridium butyricum* Ago and *Myxococcus xanthus* Ago [33].

## 3. Applications of Argonaute-Based Nucleic Acid Detection

### 3.1. Application in Bacterial Detection

One of the most critical factors in the emergence of severe diseases that pose significant risks to human wellbeing and survival is pathogenic bacteria. Typical pathogens, such as *Staphylococcus aureus, Helicobacter pylori, Klebsiella, Escherichia coli, Salmonella*, and *Listeria monocytogenes*, can be easily transmitted through food, soil, water, and air [34,35]. Jang et al. developed the ANCA detection system, making it possible to detect pathogens in one step without the amplification of carbapenemase-producing *Klebsiella pneumoniae* (CPKP). This system directly identifies CPKP in blood or urine without needing to extract or amplify nucleic acids. Additionally, these researchers fabricated 3D nanomaterials to collect bacteria from environmental samples (e.g., skin, desktops, scissors, gloves, doorknobs, and tweezers), followed by CPKP detection using the ANCA technology. The results demonstrated detection limits of 1.87 fM for CPKP. In addition, the authors demonstrated that a similar ANCA method allows other strains of *Klebsiella* pneumonia-producing imipenemase (IMP, LOD = 178 aM), New Delhi metallo-beta-lactamase (NDM, LOD = 120 aM), Verona integron-encoded metallo-beta-lactamase (VIM, LOD = 529 aM), and oxacillinase-48 (OXA-48, LOD = 144 aM) to be detected [27]. Zhao et al. created a fluorescent biosensor using phage and CbAgo to detect live *Salmonella typhimurium*, eliminating the need for complicated DNA extraction and amplification. This detection system achieves the highly sensitive detection of *S. typhimurium*, with a detection limit of 40.5 CFU/mL and a linear range of 50–10^7^ CFU/mL [36]. Li et al. established the Ago-DNAzyme cascade detection system, STAND, for the detection of methicillin-resistant *Staphylococcus aureus*. This system enables the simultaneous detection of the nuc and mecA genes in a single reaction with a sensitivity of 1 CFU/mL and a dynamic range of 1–10^8^ CFU/mL [37]. Furthermore, Li et al. integrated tag-specific primers and exonuclease I with PfAgo to develop the NOTE-Ago detection method based on the PfAgo system. Using this method, they successfully detected *Salmonella* typhi and *Staphylococcus aureus* contamination in complex real-world samples such as orange juice and eggs [38].

### 3.2. Application in Virus Detection

Throughout history, viruses have co-evolved with humans, resulting in numerous outbreaks and epidemics that have claimed millions of lives [39]. Notable examples include the influenza virus, SARS coronavirus, Ebola virus, Zika virus, and Middle East Respiratory Syndrome virus, all of which have exerted considerable strain on healthcare systems and the worldwide economy [1,40]. In the early phases of a pandemic, it is crucial to quickly and precisely detect the pathogenic virus using methods that are both sensitive and specific, enabling the swift application of control measures [41,42]. Ye et al. developed a novel multiplex rapid Argonaute-based nucleic acid detection platform, named MULAN, which allows for high specificity and the rapid portable detection of mixed samples containing Influenza A virus, Influenza B virus, and the novel coronavirus. This platform achieves a total reaction time of approximately 45 min, with a minimum detectable viral RNA concentration of 320 copies/mL [28]. Additionally, He et al. have successfully employed a multiplex nucleic acid detection method mediated by PfAgo to simultaneously identify HPV virus subtypes (HPV11, HPV16, HPV18, HPV33, and HPV45) in patient samples [23]. Wang et al. designed the PfAgo nucleic acid detection technology to recognize single-base mutations between the wild-type genome and its mutant, effectively distinguishing the novel coronavirus from other coronaviruses and achieving multi-channel detection within 70 min [43]. Kong et al. developed a graphene field-effect transistor platform mediated by Argonaute protein (Ago-GFET) capable of identifying single-nucleotide mutations in viral RNA, whose sensitivity can reach 94.7% and specificity can reach 93.3%. The author measured different virus samples (Influenza A, Influenza B, and Middle East Respiratory Syndrome coronavirus) using Ago-GFET and successfully identified the wild type and the mutant type [44].

### 3.3. Application in Fungal Detection

Foodborne pathogenic microorganisms, agricultural and veterinary drug residues, mycotoxins, antibiotic residues, and viruses can lead to food safety issues and human diseases. Among these, mycotoxins contaminating food can damage human organs by disrupting cellular structures and inhibiting protein synthesis, causing toxic symptoms and posing long-term threats to human health. A detection platform was developed by Lu et al., utilizing mesophilic Argonaute and millimeter-sized polystyrene spheres, to identify multiple non-nucleic acid targets simultaneously in one tube. This system can identify mycotoxins in food, including ochratoxin A, aflatoxin B1, and zearalenone, with linear detection ranges of 10–500 ng/mL, 5–200 ng/mL, and 5–100 ng/mL, respectively [45]. Li et al. established a microsphere resistance counting platform based on polystyrene microsphere size-based encoding and CbAgo decoding. This platform utilizes a target DNA-modified polystyrene microsphere encoding system combined with microhole resistance counting. Subsequently, the guide DNA-activated CbAgo protein’s accurate recognition and cleavage abilities allow for the decoding of the encoded microsphere system, with variations in polystyrene microsphere concentration acting as the signal readout. The platform achieves the multiplex detection of three mycotoxins—aflatoxin B1, ochratoxin A, and deoxynivalenol—with a sensitivity range spanning over four orders of magnitude, reaching picogram-per-milliliter levels [46].

### 3.4. Application in Mycoplasma and Parasite Detection

The actual application scope of Ago is expanding beyond the aforementioned detection targets. Due to the absence of a cell wall, mycoplasma is not affected by antibiotics that target cell walls, including β-lactams, fosfomycin, and glycopeptides [47]. *Mycoplasma synoviae* (MS) is a crucial pathogen that affects laying hens, causing considerable economic losses in the poultry industry. Zhao et al. developed an MS detection technique that integrates PfAgo with RPA. By meticulously designing and screening RPA primers and PfAgo-gDNA, and optimizing the conditions, the amplification and detection can be finished in 40 min, with results visible under UV light. With a sensitivity of 2 copies/mL, MS detection shows no cross-reaction with other pathogens according to specificity results. In an analysis of 31 clinical samples, this method’s results were in complete agreement with qPCR. This method does not require complex instruments and equipment, is simple to operate, and offers a dependable and user-friendly method to detect MS on location [48].

*Entercytosozoon hepatopenaei* (EHP) is a cell-parasitizing microorganism that has significantly impacted the global shrimp farming industry by causing a reduced appetite, delayed growth, and decreased production [49]. The dense spore wall of EHP enables it to resist drugs. Currently, apart from measures such as culling infected shrimp and the disinfection of the aquaculture environment, no effective treatment options exist for controlling EHP infections. Yang et al. developed a nucleic acid detection method that integrates RPA with PfAgo, allowing for on-site EHP detection in shrimp farms. This method exhibits excellent specificity, achieving single-copy sensitivity, and the entire process, including sample handling, is completed within 1.5 h. It requires only minimal laboratory support, making it highly suitable for field environments. This marks the initial use of PfAgo for diagnosing infectious diseases within the seafood supply chain [50]. This technology is important for preventing EHP outbreaks and serious future consequences, achieving the early, precise, sensitive, economical, and timely detection of EHP.

## 4. Conclusions

Nucleic acid testing technologies feature a variety of technical approaches within the realm of pathogen diagnosis, each possessing distinct attributes in a complementary manner. qPCR is regarded as the traditional “gold standard”, predominantly utilized in clinical laboratories due to its well-established, standardized procedures and extensive dynamic detection range. Nonetheless, qPCR is heavily reliant on specialized instrumentation and is limited in achieving single-base resolution. In contrast, the CRISPR-Cas system offers remarkable single-base recognition capabilities and signal amplification through programmable gRNA design and cis/trans cleavage activities. Despite these advantages, its reliance on PAM/PFSs sequences and constraints in multiplex detection limits its broader applicability; however, the emerging nucleic acid detection system based on Ago proteins has high flexibility and wide applicability in pathogen detection applications and broad used in pathogen detection applications. Its accuracy and sensitivity have been confirmed in multiple pathogen detection contexts. Liu et al. constructed an A-Star system based on Ago proteins, which can specifically amplify SNV alleles down to 0.01% with an enrichment efficiency surpassing 5500-fold [24]. This approach, while only validated for tumor gene mutation detection, demonstrates that the nucleic acid detection method using Ago proteins could be applied to low-load nucleic acid detection, facilitating precise detection. To address the requirements of multi-pathogen detection, Ago proteins can facilitate the simultaneous identification of multiple targets via targeted design, thereby offering an efficient diagnostic solution for cases involving mixed or co-infections. In response to the challenge of interference from complex samples (e.g., blood, environmental samples, or food matrices), the nucleic acid recognition capability of Ago proteins in conjunction with pretreatment technologies can effectively mitigate background interference and enhance the reliability of detection. Moreover, by combining the specific recognition and precise cleavage capabilities of the Ago system with the efficient amplification advantages of isothermal amplification technology, the developed nucleic acid detection system enables rapid signal acquisition and detection. This leads to swift, specific, and highly sensitive detection in POCT as well as resource-limited settings. This technology offers robust technical support for the “early detection, early isolation, and early treatment” prevention and control strategy, thereby facilitating the practical implementation of precision medicine in infectious disease management. In addition, by systematically comparing the advantages and disadvantages of various detection technologies, this approach can provide a scientific basis for the selection of detection methods in different application scenarios and also point out the direction for the integrated development of the next generation of nucleic acid detection technologies (Table 2).

While the Ago protein represents a more recent advancement compared to the CRISPR/Cas system in the field of biosensing, its development is progressing rapidly. It demonstrates catalytic activity in both in vivo and in vitro environments, characterized by specific recognition and efficient cleavage, thereby indicating significant potential for application in biosensing platforms. Wang et al. developed a phage-assisted Argonaute-mediated fluorescence (PDAAF) biosensor triggered by the synergy of DNA and ATP. This sensor features phages to specifically recognize and lyse *Salmonella*, releasing DNA and ATP to collaboratively trigger the Argonaute-mediated fluorescent biosensor. The linear detection range of this sensor is 10^2^ CFU/mL to 10^7^ CFU/mL, and the LOD is 20 CFU/mL. This method enables the ultra-sensitive detection of live *Salmonella* bacteria without the need for DNA extraction and amplification steps [51]. Currently, it is difficult to perform multiple detections with this method, and this may be achieved in future research by combining this method with microfluidic technology. The Ago-mediated biosensor exhibits an enhanced fluorescence signal, facilitating ultra-sensitive detection without the need for amplification. The integration of platforms such as Ago-protein-mediated nucleic acid detection, biosensors, and microfluidics holds the potential to substantially improve the performance and broaden the application scope of pathogen detection. Future advancements in high-precision, scenario-adaptive intelligent detection systems are anticipated to be driven by the enhancement of nanomaterials, the optimization of probe design through artificial intelligence, and the development of multimodal combinations (e.g., the AGE-CRISPR-sensor triple system). These innovations are poised to provide novel tools for the prevention and control of infectious diseases.

Currently, detection system outcomes are predominantly assessed through fluorescence intensity patterns. Thus, incorporating different signal output modalities, such as surface-enhanced Raman spectroscopy [52], electrochemical methods [53], and nanopore sensing platforms, which can enhance signal recognition capabilities and further improve detection sensitivity, is suggested. Additionally, non-amplification detection methods are gaining research attention due to their advantages of time efficiency, operational simplicity, cost-effectiveness, and their reduced environmental impact [33,54,55]. Without the presence of guiding DNA, Ago proteins can easily form Apo, leading to non-specific cleavage reactions in dsDNA [27,56]. To address this issue, the Ago proteins’ concentration should be carefully controlled to avoid excessively high levels. While higher concentrations of the Ago protein generally favor more enhanced reaction kinetics, identifying an APO-free form of Ago proteins in the future could fundamentally resolve these two challenges and enhance detection accuracy. Additionally, for CRISPR/Cas systems, multiple detection strategies that do not require target amplification have been developed [57]. However, analogous research for Ago proteins remains a significant challenge. The success of the CRISPR/Cas system could serve as a guiding framework for the development of biosensors based on Ago proteins for pathogen detection and other targets. Looking ahead, with advancements in Ago protein engineering and the integration of automated detection platforms, this technology is anticipated to further improve detection efficiency, reduce costs, and facilitate the advancement of precision medicine and public health monitoring systems.

## Figures and Tables

**Table 1:** Characteristics of Argonaute-based nucleic acid detection.

Detection Method	Argonaute	Guide Oligos	Amplification	Target	Sensitivity	Multiple Detection	Mutation Site	Time	Characteristics	Reference
PAND	pfAgo	gDNA	PCR amplification	DNA	1.6 aM	yes	yes	2 h	It can achieve multiple detections and mutation detections (background DNA with a concentration as low as 0.1%).	[23]
A-Star	pfAgo	gDNA	PCR amplification	DNA	34 ng	yes	yes	30 min	The ultra-high efficiency enrichment of low-abundance mutant genes (quantifying rare mutations of 0.01%, with an enrichment efficiency as high as 5500 times) requires the detection of enriched sequences in combination with technologies such as gene sequencing.	[24]
USPCRP	pfAgo	gDNA	ultrashort PCR	DNA	10 aM	no	yes	50 min	Nucleic acid detection with high sensitivity, high specificity, and single-base resolution.	[25]
TEAM	TtAgo	gDNA	exponential isothermal amplification	miRNA	1 aM	yes	yes	30–35 min	Nucleic acid detection with high sensitivity and single-base resolution.	[26]
ANCA	TtAgo	gDNA	no	DNA	1.87 fm	no	no	1 h	No sample processing or nucleic acid extraction is required. Blood samples and urine samples can be directly detected. The system composition is simple, and the operation is convenient. Only one step of the reaction is needed to complete the detection, reducing the risk of cross-contamination.	[27]
MULAN	pfAgo	gDNA	RT-LAMP	RNA	320 copies/mL	yes	yes	45 min	Realizes the highly sensitive, highly specific, and rapid portable detection of virus samples.	[28]
PASS	CbAgo	gDNA	no	DNA	<102 CFU/mL	yes	no	54 min	This system realizes amplification-free multi-pathogen detection at ambient temperature. The method integrates the image-processing technology of deep learning, and the results show high accuracy and high sensitivity.	[29]

**Table 2:** Advantages and disadvantages of nucleic acid testing techniques.

Method	Advantages	Disadvantages
qPCR	The “gold standard” for nucleic acid detection; Accurate quantification with a broad dynamic range; Mature technology with high standardization.	Requires sophisticated instrumentation, dedicated laboratory facilities, and trained personnel; Limited single-nucleotide polymorphism discrimination capability.
CRISP-Cas	Programmability, high sensitivity, and single-base resolution; Cas enzymes exhibit both cis- and trans-cleavage activities, enabling enhanced detection sensitivity; Diverse output signal modalities, including fluorescence, colorimetry, and electrochemistry; Numerous studies have demonstrated amplification-free CRISP-Cas detection approaches.	Sequence recognition requires specific PAM/PFS motifs; Incapable of multiplex detection in a single-tube reaction.
Argonaute	Programmability, high sensitivity, and single-base resolution; Short gDNA length enables facile design and enhanced stability; Ago proteins lack trans-cleavage activity and perform sequence-specific cleavage only, ensuring superior targeting precision; Single-enzyme implementation enables multiplex detection in one-pot reactions; Universal applicability across diverse scenarios (e.g., resource-limited settings and POCT).	The signal output mode is relatively simple (fluorescence); Amplification-free Ago detection systems remain understudied; Available Ago variants are predominantly thermophilic, with limited development of mesophilic orthologs.
